# Genome-wide search identifies a gene-gene interaction between 20p13 and 2q14 in asthma

**DOI:** 10.1186/s12863-016-0376-3

**Published:** 2016-07-07

**Authors:** William Murk, Andrew T. DeWan

**Affiliations:** Department of Chronic Disease Epidemiology, Yale School of Public Health, 60 College St., New Haven, CT 06510 USA

**Keywords:** Asthma, Gene-gene interaction, Epistasis, ADAM33, GLI2

## Abstract

**Background:**

Many studies have attempted to identify gene-gene interactions affecting asthma susceptibility. However, these studies have typically used candidate gene approaches in limiting the genetic search space, and there have been few searches for gene-gene interactions on a genome-wide scale. We aimed to conduct a genome-wide gene-gene interaction study for asthma, using data from the GABRIEL Consortium.

**Results:**

A two-stage study design was used, including a screening analysis (*N* = 1625 subjects) and a follow-up analysis (*N* = 5264 subjects). In the screening analysis, all pairwise interactions among 301,547 SNPs were evaluated, encompassing a total of 4.55 × 10^10^ interactions. Those with a screening interaction *p*-value < 10^−5^ were evaluated in the follow-up analysis. No interaction selected from the screening analysis met strict statistical significance in the follow-up (*p*-value < 1.45 × 10^−7^). However, the top-ranked interaction (rs910652 [20p13] × rs11684871 [2q14]) in the follow-up (*p*-value = 1.58 × 10^−6^) was significant in one component of a replication analysis. This interaction was notable in that rs910652 is located within 78 kilobases of *ADAM33*, which is one of the most well studied asthma susceptibility genes. In addition, rs11684871 is located in or near *GLI2*, which may have biologically relevant roles in asthma.

**Conclusions:**

Using a genome-wide approach, we identified and found suggestive evidence of replication for a gene-gene interaction in asthma involving loci that are potentially highly relevant in asthma pathogenesis.

**Electronic supplementary material:**

The online version of this article (doi:10.1186/s12863-016-0376-3) contains supplementary material, which is available to authorized users.

## Background

Asthma is a chronic disease with a strong genetic component in its etiology, having heritability estimates ranging from 25 to 73 % [1, 2]. However, the specific genetic factors that underlie this heritability remain unclear, as most factors identified thus far tend to have weak effect sizes and are poorly replicated across studies [3, 4]. Gene-gene interaction, the phenomenon where the phenotypic effect of a variant at one genetic locus depends on variants at other loci [5, 6], is one possible explanation for this “missing heritability” and the lack of replication of genetic effects for many complex diseases [7–9]. Thus, the identification of gene-gene interactions could enable a greatly improved understanding of the genetic etiology of asthma.

More than 45 studies have attempted to identify gene-gene interactions in asthma, with over 190 significant interactions reported thus far [10]. However, these studies have tended to use candidate gene approaches to narrow the genetic space in which to search for interactions, typically focusing on loci that have been reported to show significant marginal effects (i.e., effects that manifest when loci are assessed individually). To our knowledge, only one asthma association study involving a genome-wide search for gene-gene interactions has been published, with null findings [11]. Although this may be due in part to the lower likelihood of publication of null findings, it appears that the search space of pairwise interactions has been sparsely explored in asthma.

In order to identify interactions involving loci that might not otherwise be considered, we aimed to conduct a genome-wide search for gene-gene interactions in asthma susceptibility. Data from the GABRIEL Consortium genome-wide association study of asthma [12] were obtained for this purpose, which included a total of 4186 cases and 3916 controls. To maintain power in detecting an interaction, we used a two-stage screening and follow-up approach. In this design, the screening stage was used to conduct the exhaustive genome-wide search for interactions, and the follow-up stage was used to assess all interactions with a *p*-value < 10^−5^. In addition, a preliminary attempt at replication was made for the most significant interaction identified from the follow-up.

## Methods

### Study datasets

All data in the current study were obtained from the GABRIEL Consortium [12] (European Genome-phenome Archive Study ID: EGAS00000000077). Approval for the use of this data was obtained from the Gabriel Consortium Data Access Committee. Data from 12 of the GABRIEL study centers were included (Table 1). In this paper, we refer to any dataset particular to an individual study center as a “sub-study” dataset. Case/control status, sex, and genotype information were available, while age and all other information were not available. In general, cases were subjects with self-reported doctor-diagnosed asthma, although some sub-studies had alternative definitions (Table 1). In particular, the AUGOSA and SEVERE sub-studies involved subjects with severe asthma. The MAS, AUGOSA, and SEVERE sub-studies only had cases available. All subjects were genotyped on the Illumina Human610 quad array (*N* = 582,892 single nucleotide polymorphisms [SNPs]).

**Table 1:** Sub-studies included from the GABRIEL Consortium

Study component	Sub-study	Reference	Country of origin	Case definition	Control definition	N, cases	N, controls
Screening	GABRIEL-AS	[28]	Austria, Germany, Switzerland	Ever had doctor-diagnosed asthma or had asthmatic bronchitis at least twice (self-report).	Did not ever have doctor-diagnosed asthma and no asthmatic bronchitis diagnosed at least twice (self-report).	802	823
Follow-up	BAMSE	[30]	Sweden	Ever had doctor-diagnosed asthma (self-report).	Did not have a history of asthma or other allergic diseases (self-report).	226	235
	BUSSELTON	[31]	Australia	Ever had doctor-diagnosed asthma (self-report).	Did not ever have doctor-diagnosed asthma (self-report).	520	685
	EGEA	[32]	France	Ever had asthma attacks (self-report).	Did not ever have asthma attacks (self-report).	120	444
	KMSU	[33]	Russia	Asthma diagnosed on the basis of symptoms (recurrent cough, wheezing, or dyspnea), airway obstruction reversibility, or airway methacholine hyper-responsiveness.	No symptoms or history of allergic disease, normal total serum IgE, normal pulmonary function.	285	261
	PIAMA	[34]	The Netherlands	Ever had doctor-diagnosed asthma (self-report).	Did not have a history of asthma or other allergic diseases (self-report).	174	187
	SAPALDIA	[35]	Switzerland	Ever had asthma (self-report).	Did not ever have asthma (self-report).	581	880
	UFA	N/A	Russia	Asthma diagnosed on the basis of clinical examination, family and medication history, and lung function tests.	No symptoms or history of asthma or other pulmonary disease, no symptoms or history of atopy, no first-degree relatives with a history of asthma or atopy.	333	333
					Sub-total (Follow-up)	2239	3025
Replication	AUGOSA	[36]	United Kingdom	Severe asthma (determined based on clinical assessments and control of symptoms)	N/A	625	0
	KARELIA	[37]	Finland, Russia	Ever had doctor-diagnosed asthma (self-report).	Did not ever have doctor-diagnosed asthma (self-report).	57	68
	MAS	[38]	Germany	Ever had doctor-diagnosed asthma.	N/A	173	0
	SEVERE	[39]	United Kingdom	Severe asthma (determined based on clinical assessments and control of symptoms)	N/A	290	0
					Sub-total (Replication)	1145	68
					Total (all)	4186	3916

More complete case/control definitions may be available in the indicated references or the supplement to Moffatt et al. [12]. Subject counts reflect final numbers for inclusion, after quality control procedures were applied

### Quality control (QC)

Subjects were excluded if any of the following were true: having a subject call rate < 95 %; having an ambiguous SNP-estimated sex (X-chromosome homozygosity, F, between 0.2 and 0.8); being flagged for removal due to relatedness with other subjects; and being flagged as a principal components outlier. Subject relatedness was determined by calculating pi-hat (proportion identical by descent) between every pair of subjects; one randomly chosen subject of every pair with pi-hat ≥ 0.20 was flagged for removal. Principal components analysis was performed using EIGENSTRAT; a subject was flagged as an outlier if he or she had a value >6 standard deviations on the top 10 components. Both subject relatedness and principal components analysis were assessed in linkage-disequilibrium (LD) pruned datasets (based on pairwise SNP correlations; r^2^ threshold of 0.5).

SNPs were excluded if any of the following were true: unable to convert from hg18 to hg19 reference genome coordinates; having a SNP call rate <98 % in either cases or controls (for MAS, AUGOSA, and SEVERE, only cases were considered); and having a test for deviation from Hardy-Weinberg equilibrium (HWE) with *p*-value < 10^–4^ among controls (for MAS, AUGOSA, and SEVERE, the criteria was *p*-value < 10^−7^ among cases).

All QC procedures were performed within each sub-study individually. QC exclusion counts and final subject characteristics are shown in Additional file 1: Tables S1, S2 and S3.

### Population stratification

To check for test statistic inflation due to population stratification or other sources, quantile-quantile (QQ) plots of observed vs. expected-under-the-null *p*-values were evaluated. This was performed for both marginal effect *p*-values and interaction *p*-values, within each sub-study (for case-only studies, only interaction QQ plots were evaluated). *P*-values from the follow-up interaction meta-analysis were also examined via QQ plots. Little or no inflation was noted (Additional file 1: Figure S1). At most, a minor amount of inflation was noted for UFA in the marginal effects, but this did not appear to manifest in the interaction effects.

### Analytical methods

#### Screening analysis

The largest sub-study available, GABRIEL-AS (GABRIEL Advanced Surveys), was used as a screening dataset. All pairwise interactions between SNPS with minor allele frequency (MAF) ≥ 0.20, only including chromosomes 1 through 22, were analyzed for the outcome of asthma. This included *N* = 301,547 SNPs and *N* = 4.55 × 10^10^ interactions. The search space was not trimmed for LD, and thus a portion of these interactions was likely statistically redundant. The analysis was performed using the logistic regression (−lr) function in the CASSI Genome-Wide Interaction Analysis Software package, due to its computational speed advantages when performing epistasis (gene-gene interaction) analyses (version 2.50; https://www.staff.ncl.ac.uk/richard.howey/cassi/index.html). All interactions with a *p*-value < 10^−5^ in this analysis were tested again using the --epistasis function in PLINK (version 1.07; http://pngu.mgh.harvard.edu/~purcell/plink/) [13]. This was done in order to confirm that interactions met the same *p*-value threshold using both methods, since minor differences in *p*-values between the methods were noted. All interactions with a *p*-value < 10^−5^ from both methods (*N* = 345,034 interactions) were included for the follow-up analysis. At this significance level, the GABRIEL-AS study has greater than 90 % power to detect interactions with an interaction odds ratio (OR) 2.5 or more, for SNPs with MAF 0.20 or more (for additional power estimates, see Additional file 1: Table S4).

#### Follow-up analysis

The seven next-largest studies containing both cases and controls, including BAMSE, BUSSELTON, EGEA, KMSU, PIAMA, SAPALDIA, and UFA, were used as the follow-up datasets. All interactions that passed the screening analysis were analyzed in each of these datasets, individually, using the --epistasis function in PLINK. Each such analysis involved a logistic regression model that included a main effect variable for each SNP and an interaction variable (defined as the product of the main effects variables), with each SNP variable coded additively (taking on the values 0, 1, or 2, depending on the number of copies of the non-referent allele present). Asthma (log odds of being a case) was the outcome. All studies used the same non-referent allele for each SNP; this was the minor allele among controls in GABRIEL-AS. Interaction *p*-values originated from a test of the null hypothesis that the interaction variable parameter is equal to zero, after adjusting for the main effects. Gene-gene interaction is represented in these models as deviation from additivity of combined SNP effects on the log-odds scale of disease. SAS version 9.3 (SAS Institute; Cary, NC) was used to obtain full parameter estimates (i.e., including both main effects and interaction terms) of the most significant interactions. Parameter estimates were meta-analyzed via fixed effects or random effects models, across the *N* = 7 follow-up studies. Heterogeneity was assessed via calculations of I^2^ and the Q test. If an interaction had an I^2^ < 50 % and a Q test *p*-value ≥ 0.10, then fixed effects estimates were used; otherwise, random effects estimates were used. Meta-analyses were performed using R version 3.11 (http://www.r-project.org) and the package “meta”, version 3.7-1 (http://cran.r-project.org/web/packages/meta/). The top ten most significant interactions from the follow-up, as ranked by meta-analysis interaction *p*-value, are listed in Additional file 1: Table S5. For the follow-up meta-analysis, statistical significance was defined as 0.05 corrected for 345,034 tests of interaction (*p* < 1.45 × 10^−7^). At this significance level, an approximate estimation of power indicates that the follow-up analysis has greater than 90 % power to detect interactions with an interaction odds ratio 1.75 or more, for SNPs with MAF 0.20 or more (for additional power estimates, see Additional file 1: Table S4).

#### Replication analysis

The smallest case-control study (KARELIA) and the case-only studies (MAS, AUGOSA, and SEVERE) were used as replication datasets. In KARELIA, the interactions chosen for replication were analyzed using logistic regression modeling, as described above. In MAS, AUGOSA, and SEVERE, the interactions were analyzed using the adjusted fast epistasis case-only test (−afe-co-only) in CASSI, which is a test of correlation between alleles at two different SNPs. Under the assumption that such a correlation does not exist in controls, the case-only test reflects a test for gene-gene interaction, since allelic co-occurrence might indicate that the alleles jointly affect disease risk [14]. More specifically, an interaction term from a logistic regression model involving binary predictors can be seen to be equivalent to the ratio of the OR of association between alleles in cases to the OR of association between alleles in controls. Therefore, if the OR of association between alleles in controls is one, then the OR of association between alleles in cases is equal to the interaction odds ratio [14]. However, findings from case-only tests must be interpreted with abundant caution, since the “no correlation among controls” assumption is potentially suspect given the effects of linkage disequilibrium [6]. Results from the four replication datasets were not meta-analyzed, due to heterogeneity in the tests used and the phenotypes assessed (AUGOSA and SEVERE involved severe asthma cases, whereas KARELIA and MAS cases were not defined based on severity), as well as due to the unidentified sources of heterogeneity that led to discordance in the directions of effects (Table 2). For the replication analysis, significance was defined as *p* < 0.05. Given the use of the case-only approach for three of the four replication datasets, results from these analyses were considered preliminary, as any significant findings will require further study using a more robust case-control approach.

**Table 2:** Model estimates of interaction between 20p13 and 2q14

	Most significant interaction in the follow-up analysis						Most significant interaction in the surrounding region					
	20p13: rs910652 (T/C) main effect		2q14: rs11684871 (G/A) main effect		rs910652 × rs11684871 interaction		20p13: rs1018491 (T/C) main effect		2q14: rs67959717 (G/A) main effect		rs1018491× rs67959717 interaction	
	OR (95 % CI)	*p*-value	OR (95 % CI)	*p*-value	OR (95 % CI)	*p*-value	OR (95 % CI)	*p*-value	OR (95 % CI)	*p*-value	OR (95 % CI)	*p*-value
Screening												
GABRIEL-AS	1.43 (1.18, 1.74)	3.22 × 10^−4^	1.36 (1.09, 1.70)	6.57 × 10^−3^	0.54 (0.42, 0.70)	1.51 × 10^−6^	1.19 (0.99, 1.44)	6.12 × 10^−2^	1.11 (0.87, 1.42)	3.90 × 10^−1^	0.78 (0.61, 0.99)	4.12 × 10^−2^
Follow-up												
BAMSE	0.62 (0.42, 0.91)	1.58 × 10^−2^	0.55 (0.36, 0.83)	4.12 × 10^−3^	2.05 (1.26, 3.32)	3.68 × 10^−3^	0.76 (0.52, 1.10)	1.40 × 10^−1^	0.60 (0.38, 0.95)	2.99 × 10^−2^	1.52 (0.99, 2.33)	5.63 × 10^−2^
BUSSELTON	0.81 (0.65, 1.02)	6.84 × 10^−2^	0.78 (0.60, 1.02)	6.55 × 10^−2^	1.35 (1.03, 1.78)	2.83 × 10^−2^	0.69 (0.56, 0.87)	1.36 × 10^−3^	0.63 (0.47, 0.83)	1.28 × 10^−3^	1.73 (1.31, 2.29)	1.11 × 10^−4^
EGEA	0.77 (0.52, 1.14)	1.93 × 10^−1^	0.88 (0.55, 1.42)	6.01 × 10^−1^	1.17 (0.68, 2.00)	5.69 × 10^−1^	0.96 (0.66, 1.38)	8.07 × 10^−1^	1.04 (0.61, 1.77)	8.84 × 10^−1^	0.94 (0.57, 1.55)	8.14 × 10^−1^
KMSU	1.03 (0.72, 1.47)	8.74 × 10^−1^	0.96 (0.68, 1.37)	8.35 × 10^−1^	1.01 (0.66, 1.55)	9.66 × 10^−1^	0.84 (0.60, 1.17)	3.04 × 10^−1^	0.72 (0.50, 1.06)	9.45 × 10^−2^	1.50 (1.02, 2.20)	4.01 × 10^−2^
PIAMA	0.89 (0.60, 1.33)	5.78 × 10^−1^	0.86 (0.55, 1.33)	4.83 × 10^−1^	1.46 (0.90, 2.37)	1.25 × 10^−1^	0.65 (0.44, 0.97)	3.67 × 10^−2^	0.62 (0.37, 1.04)	6.80 × 10^−2^	2.04 (1.23, 3.40)	5.94 × 10^−3^
SAPALDIA	0.91 (0.74, 1.11)	3.63 × 10^−1^	0.82 (0.64, 1.05)	1.10 × 10^−1^	1.30 (1.01, 1.69)	4.50 × 10^−2^	1.04 (0.85, 1.26)	7.27 × 10^−1^	0.83 (0.63, 1.09)	1.81 × 10^−1^	1.22 (0.94, 1.57)	1.32 × 10^−1^
UFA	0.66 (0.47, 0.91)	1.03 × 10^−2^	0.62 (0.44, 0.87)	5.46 × 10^−3^	2.03 (1.35, 3.03)	5.99 × 10^−4^	0.81 (0.60, 1.09)	1.65 × 10^−1^	0.68 (0.48, 0.98)	3.61 × 10^−2^	1.54 (1.05, 2.24)	2.55 × 10^−2^
Meta-analysis	0.82 (0.74, 0.92)	5.84 × 10^−4^	0.77 (0.68, 0.88)	6.06 × 10^−5^	1.40 (1.22, 1.61)	1.58 × 10^−6^	0.84 (0.75, 0.93)	1.23 × 10^−3^	0.71 (0.62, 0.82)	1.89 × 10^−6^	1.45 (1.27, 1.66)	5.78 × 10^−8^
Heterogeneity	*I* ^2^ = 11.2 %; *Q* = 0.344		*I* ^2^ = 9.3 % *Q* = 0.358		*I* ^2^ = 30.8 %, *Q* = 0.193		*I* ^2^ = 37.0 %; *Q* = 0.146		*I* ^2^ = 0.0 %; *Q* = 0.581		*I* ^2^ = 27.1 %; *Q* = 0.221	
Replication												
KARELIA	1.18 (0.57, 2.48)	6.56 × 10^−1^	1.16 (0.54, 2.47)	7.09 × 10^−1^	0.58 (0.24, 1.44)	2.41 × 10^−1^	1.56 (0.76, 3.21)	2.25 × 10^−1^	0.65 (0.28, 1.55)	3.32 × 10^−1^	0.93 (0.38, 2.30)	8.80 × 10^−1^
	Case-only OR (95 % CI)				Case-only *p*-value		Case-only OR (95 % CI)				Case-only *p*-value	
MAS	1.37 (1.01, 1.86)				4.20 × 10^−2^		0.96 (0.69, 1.34)				8.08 × 10^−1^	
AUGOSA	1.18 (0.98, 1.42)				7.34 × 10^−2^		0.99 (0.83, 1.19)				9.42 × 10^−1^	
SEVERE	0.79 (0.59, 1.07)				1.31 × 10^−1^		0.75 (0.55, 1.01)				6.10 × 10^−2^	

See the Methods section for model descriptions. Major/minor alleles, respectively, are indicated next to SNP RSIDs. OR: odds ratio. CI: confidence interval. *P*-value: *p*-value for the test of the null hypothesis that the respective parameter estimate is equal to zero. Meta-analysis: fixed effects meta-analysis for the follow-up studies. I^2^: percentage of effect variability due to heterogeneity between studies. Q: *p*-value from a Q test for heterogeneity. The case-only ORs reflect correlation between alleles of the two SNPs, and the *p*-value is for the test of the null hypothesis that no such correlation exists. Characteristics of the SNPs are shown in Additional file 1: Tables S6 and S7

#### Assessment of interactions surrounding the top hit in the follow-up analysis

Variants located within intervals spanning 100 kilobases (Kb) upstream and downstream of rs11684871 (chromosome 2) and rs910652 (chromosome 20) were imputed using IMPUTE2 (https://mathgen.stats.ox.ac.uk/impute/impute_v2.html) and 1000 Genomes reference haplotype panels (Phase 1 integrated variant set release v3) [15]. This was done within each sub-study individually, after quality control procedures had been applied. The buffer size was set as 250 Kb, and reference SNPs with MAF < 0.01 among 1000 Genomes subjects of European descent were filtered (ignored) during the imputation. In the screening and follow-up datasets, all QC-passed directly genotyped SNPs within the interval specified above, and all imputed SNPs with certainty ≥ 0.80 and info ≥ 0.50, were included for analysis. SNPs were excluded from analysis if they had a MAF < 0.10 (based on frequencies in GABRIEL-AS controls). Pairwise interactions between chromosome 2 and 20 SNPs were assessed using the PLINK --epistasis function (*N* = 124,502 interactions). Statistical significance for the follow-up studies in analyzing interactions involving these regions was set as 1.06 × 10^−7^ (0.05 corrected for 345,034 + 124,502 = 469,536 interactions). For replication in the case-only sub-studies, the CASSI case-only analysis was used, as described above. Since the imputation quality was high (median concordance across the 12 sub-studies: 95.6 for chromosome 2 SNPs, 96.5 for chromosome 20 SNPs), SNPs were analyzed using rounded genotypes, where the highest probability genotype was called as 1 if its probability was ≥ 0.90, and the other two genotypes were called as 0; if no probability was ≥ 0.90, then all three genotypes were set as missing.

### Regulatory annotation

**Fig. 1:**
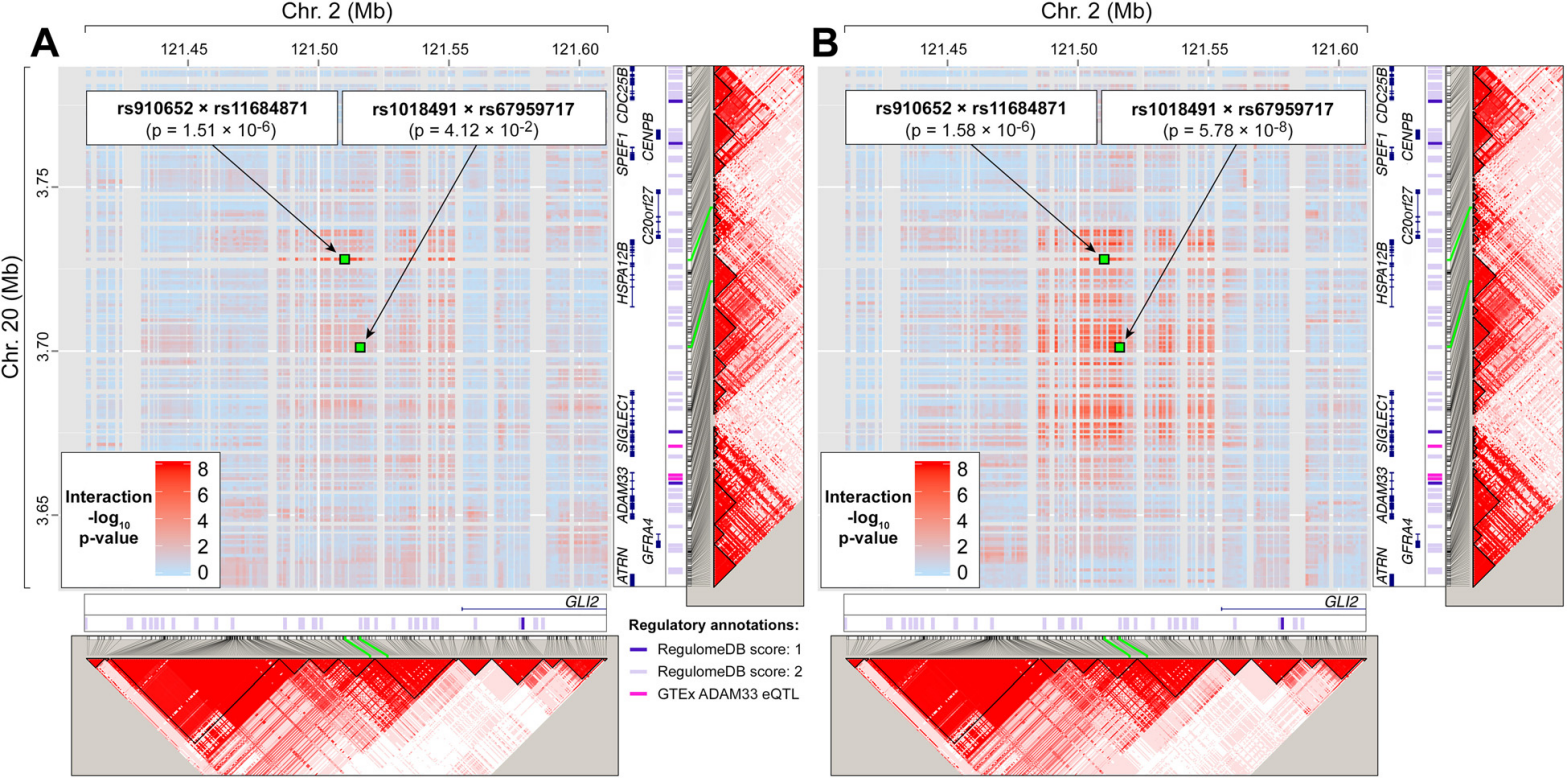
Gene-gene interactions between 20p13 and 2q14. *P*-values for interactions between variants within 0.1 megabases (Mb) of rs910652 and rs11684871 are represented. Gene annotations are from LocusZoom (http://locuszoom.sph.umich.edu/locuszoom/); regulatory annotations are from RegulomeDB and GTEx (see the Methods section). The side plots show LD structure; stronger colors on these plots indicate higher pairwise LD as measured by D’ (generated via Haploview [http://www.broadinstitute.org/haploview]). Interaction *p*-values and LD measures from the screening dataset (**a**) and follow-up datasets (meta-analysis *p*-values) (**b**) are shown. The green lines identify correspondence between genomic positions and LD plot positions, for the highlighted variants

In order to identify any possible functional, regulatory sites within the genomic intervals surrounding the SNPs involved in the top interaction (as specified above), we used data from RegulomeDB [16] and GTEx [17]. Specifically, RegulomeDB Version 1.1 was used to identify all genomic positions having a RegulomeDB score of 1 or 2 (i.e., sites that are likely to affect transcription factor binding). GTEx Analysis Version 4 was used to identify all significant eQTL SNPs for the gene targets of *ADAM33*, *HSPA12B*, *SIGLEC1*, and *GLI2* (as determined from any tissue type). Only three eQTL SNPs, all for *ADAM33*, were found (rs609203, rs2853211, and rs554743). These annotations were used to illustrate Fig. 1.

## Results

### Screening analysis

In the screening analysis, all 4.55 × 10^10^ pairwise interactions among the 301,547 SNPs with MAF ≥ 0.20 were assessed. The most significant interaction from this analysis (rs12289833 [11q25] × rs5027694 [13q13]) had a *p*-value of 3.43 × 10^−10^. A total of 345,034 interactions with a *p*-value < 10^−5^ from the screening analysis were included for further evaluation in the follow-up analysis.

### Follow-up analysis

**Fig. 2:**
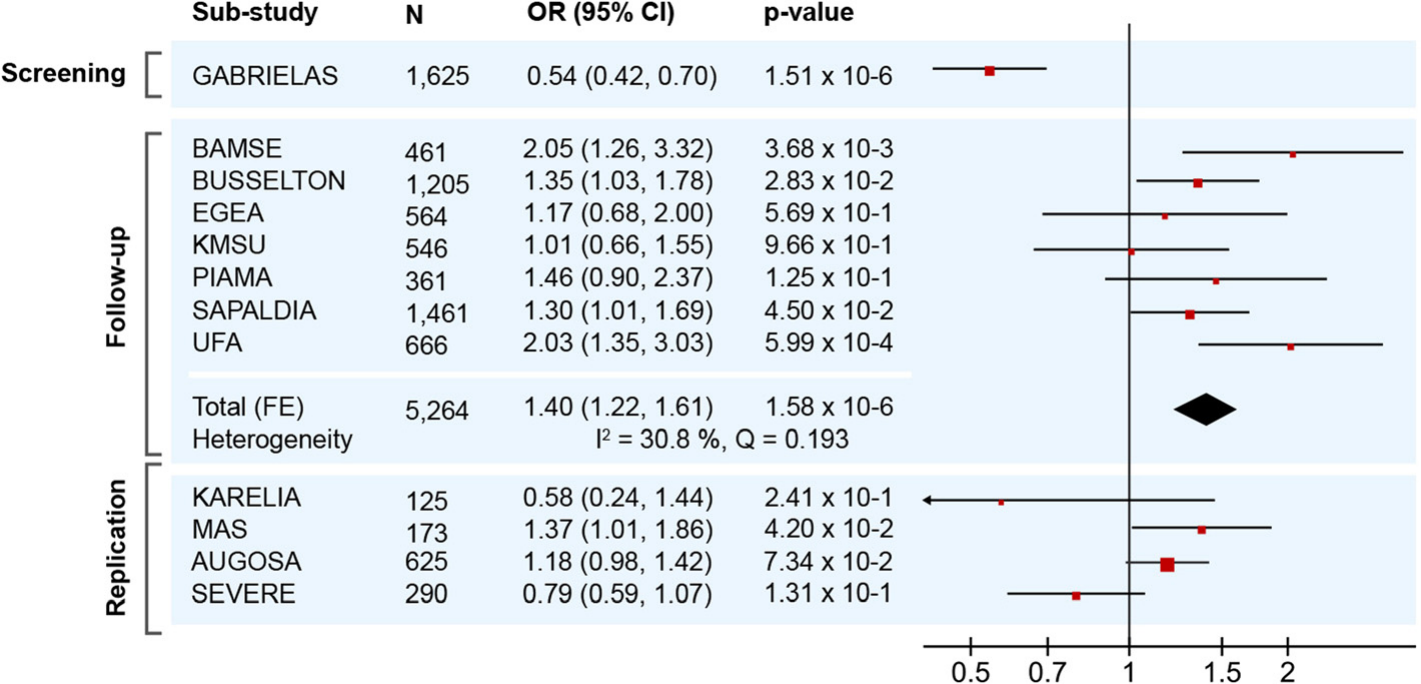
Forest plot of effect estimates for the interaction between rs910652 and rs11684871. Parts of this figure were made with RevMan 5.3 (http://tech.cochrane.org/revman)

In the follow-up analysis, no interaction exceeded the pre-specified significance threshold of *p* < 1.45 × 10^−7^ after being meta-analyzed across the seven follow-up sub-studies. The top-ranked interaction in the follow-up (rs910652 [20p13] × rs11684871 [2q14]) had a meta-analysis *p*-value of 1.58 × 10^−6^, although the interaction odds ratio was not consistent with that of the screening analysis (Fig. 2; Table 2). Interestingly, rs910652 is located approximately 78 kilobases upstream of *ADAM33*, which is one of the most well-studied susceptibility loci for asthma. The other SNP, rs11684871, is either upstream, or in an intron, of *GLI2*, depending on which splice variant is considered. Due to the possible involvement of the *ADAM33* locus in this interaction, a more detailed investigation of the surrounding pairwise chromosomal space was performed. Specifically, we imputed variants 100 kilobases upstream and downstream of each SNP, and assessed all interactions between these areas (*N* = 124,502 interactions; Fig. 1). In these areas, the strongest interaction (rs1018491 × rs67959717) had a *p*-value of 5.78 × 10^−8^, which exceeded a conservative significance threshold of 1.06 × 10^−7^ (Table 2; Fig. 1).

### Replication analysis

Next, we evaluated the rs910652 × rs11684871 and rs1018491 × rs67959717 interactions in the replication analysis (Fig. 2; Table 2). A case-only interaction analysis was performed for MAS, AUGOSA, and SEVERE, since these datasets did not contain controls. Two of these sub-studies, AUGOSA and SEVERE, involved subjects with severe asthma. The rs910652 × rs11684871 interaction was significant at *p* < 0.05 in MAS (*p* = 0.042) and approached significance in AUGOSA (*p* = 0.073). The case-only odds ratios for these studies were consistent in direction with the follow-up studies (confirmed by also performing case-only analyses in the follow-up studies; not shown). The interaction was not significant and in opposite direction in KARELIA and SEVERE. The rs1018491 × rs67959717 interaction was not significant in any replication dataset; it approached significance (*p* = 0.061) in SEVERE, although the direction was opposite that of the direction in the follow-up studies.

## Discussion

The top-ranked gene-gene interaction in the follow-up analysis (rs910652 × rs11684871) involved a SNP (rs910652) located near the *ADAM33* gene. *ADAM33* was the first gene to be linked to asthma susceptibility, and numerous studies have confirmed associations between this gene and asthma, although the specific causal variants are unclear and there are notable inconsistencies across studies [18]. Despite the fact that rs910652 is located in a flanking gene (*HSPA12B*), and does not appear to be in strong LD with *ADAM33* (Fig. 1), one possible explanation for our findings is that this SNP could be tagging a variant that influences the regulation of nearby genes. This is indirectly suggested by our observation that the strongest interaction in the surrounding region (rs1018491 × rs67959717) involves a SNP (rs1018491) that is located intergenically, closer to *ADAM33* and between *SIGLEC1* and *HSPA12B*. This SNP is 38 bases away from a genomic position (chr20:3701170; itself not imputed in the current study) that may affect transcription factor binding (RegulomeDB score: 2b; putative bound protein: TCF12). However, the rs1018491 × rs67959717 interaction was not replicated, which could suggest that the functional variant is actually located elsewhere on chromosome 20. The closest known *ADAM33* eQTL (rs609203) is in an intron of the *SIGLEC1* gene (Fig. 1), but the strongest interaction involving this SNP was of modest significance (rs609203 × rs4848623; follow-up interaction *p*-value: 0.0026; data not shown). An alternative explanation is that *HSPA12B* itself is the functional gene of the interaction on chromosome 20. This gene does not have a clear role in asthma, but one study has reported its association with lung function at the same SNP we identified (rs910652) [19].

The other gene in the identified interaction, *GLI2*, has been reported to be involved in T-cell function [20, 21] and TGFβ expression [22], which are both well-known components of asthma pathogenesis [23, 24]. In addition, this gene is known to play a role in lung development [25, 26]. Thus, it is highly plausible that *GLI2* has biological relevance in asthma. Transcriptional activation of *GLI2* depends on *SMAD3* [27], which was one of the top asthma-associated genes in the original GABRIEL study [12]. The functional interaction that underlies our observed statistical interaction is unclear, as such an interaction between *GLI2* and either *ADAM33*, *SIGLEC1*, or *HSPA12B* has not been described. However, suggestions of a biological relationship between *GLI2* and *ADAM33* can perhaps be seen in the fact that both of these genes are regulated by TGFβ [18, 27].

A limitation to our results is that the directions of effect of the identified interactions were inconsistent between the screening and follow-up analyses (Table 2). The simplest explanation for this is that the detected signal arose by chance in the screening analysis. An alternative, mechanistic explanation is that the direction of the observed gene-gene interaction depends on an unknown environmental exposure (e.g., when a subject is exposed, the gene-gene interaction is epistatically protective, and when unexposed, it is epistatically deleterious) that has differences in prevalence between the screening and follow-up studies. Although most of the included GABRIEL sub-studies are relatively similar to one another, one notable difference between the GABRIEL-AS (screening) sub-study and the others is that, in GABRIEL-AS, subjects were recruited from rural areas [28], which may have an overall protective effect on asthma [29]. It is possible that different variants on chromosome 20 are involved in this hypothetical environmental dependency, given that the gene-gene interaction signal maximizes at different chromosomal locations between the screening and follow-up studies (Fig. 1).

An additional limitation is that we relied on case-only analysis for replication. This method assumes that correlation between alleles only exists in cases (due to gene-gene interaction affecting the phenotype) and not in controls; this assumption could be violated, particularly if linkage disequilibrium exists between the SNPs. Since the interacting SNPs are on different chromosomes, we believe that this assumption is not unreasonable, but nevertheless a case-control approach is required to confirm the replication. Finally, the screening analysis was powered to detect interaction ORs of 2.50 and above (for MAF 0.2 and above at each SNP); this may be an unrealistically high effect estimate, and the study likely missed interactions of weaker effect and/or less common SNPs from the screening stage.

## Conclusions

In summary, using a genome-wide approach of screening for gene-gene interactions, we identified and found suggestive evidence of replication for an interaction in asthma (rs910652 × rs11684871), where one of the participating SNPs (rs910652) is in close proximity to *ADAM33* and the other (rs11684871) is located in or near *GLI2*. Both SNPs are highly plausible asthma risk variants, given known associations between *ADAM33* and asthma, a previously reported association between rs910652 and lung function, and the roles of *GLI2* in T-cell regulation and lung development. To our knowledge, *GLI2* has not been previously implicated in asthma susceptibility. This could be explained by our observations that the *GLI2* SNPs did not manifest any marginal effects (Additional file 1: Tables S6-S7), suggesting that these SNPs would be undetected in the traditional association study design that examines each locus individually.

Further investigation is required to confirm the putative interaction identified in the present study, as well as to identify the possible causal loci underlying the interaction. If the interaction is genuine, it could provide new insight into the biological role of *ADAM33* or its neighboring genes in asthma susceptibility, and may also emphasize *GLI2* as a new gene of investigation for asthma. This study may also inform general strategies for epistasis investigations: given that one putatively interacting locus is in a region previously associated with asthma or lung function (*ADAM33*/*HSPA12B*), and the other is highly biologically plausible (*GLI2*), this study provides evidence in support of investigational strategies that restrict the epistasis search space to features with prior disease relevance.

## Abbreviations

CI, confidence interval; Kb, kilobases; MAF, minor allele frequency; Mb, megabases; OR, odds ratio; QC, quality control; QQ, quantile-quantile; SNP, single nucleotide polymorphism

## Additional file

Additional file 1:
**Figure S1.** Quantile-quantile (QQ) plots of marginal and interaction test *p*-values. **Table S1.** Subject quality control. **Table S2.** SNP quality control. **Table S3.** Final subject characteristics. **Table S4.** Power analysis for the current study. **Table S5.** The top 10 most significant interactions in the follow-up analysis. **Table S6.** Characteristics of rs910652 and rs11684871 (SNPs involved in the most significant interaction in the follow-up analysis). **Table S7.** Characteristics of rs1018491 and rs67959717 (SNPs involved in the most significant interaction in the surrounding region of 20p13 and 2q14). (PDF 688 kb)
