# Supplementary material for: Genome-wide search identifies a gene-gene interaction between 20p13 and 2q14 in asthma
Source: BMC Genet. 2016 Jul 7;17:102. doi: 10.1186/s12863-016-0376-3 (PMC4936310; doi:10.1186/s12863-016-0376-3)
Supplement: Additional file 1: — Figure S1. Quantile-quantile (QQ) plots of marginal and interaction test p-values. Table S1. Subject quality control. Table S2. SNP quality control. Table S3. Final subject characteristics. Table S4. Power analysis for the current study. Table S5. The top 10 most significant interactions in the follow-up analysis. Table S6. Characteristics of rs910652 and rs11684871 (SNPs involved in the most significant interaction in the follow-up analysis). Table S7. Characteristics of rs1018491 and rs67959717 (SNPs involved in the most significant interaction in the surrounding region of 20p13 and 2q14). (PDF 688 kb) [file 12863_2016_376_MOESM1_ESM.pdf]

## Genome-wide search identifies a gene-gene interaction between 20p13 and 2q14 in asthma

William Murk, MPH and Andrew T. DeWan, PhD*
Department of Chronic Disease Epidemiology, Yale School of Public Health, 60 College St., New Haven, CT 06510, USA.
*Corresponding author. Email: andrew.dewan@yale.edu, Tel: 203-785-3528

## ADDITIONAL FILE 1

---

**Figure S1. Quantile-quantile (QQ) plots of marginal and interaction test p-values.**

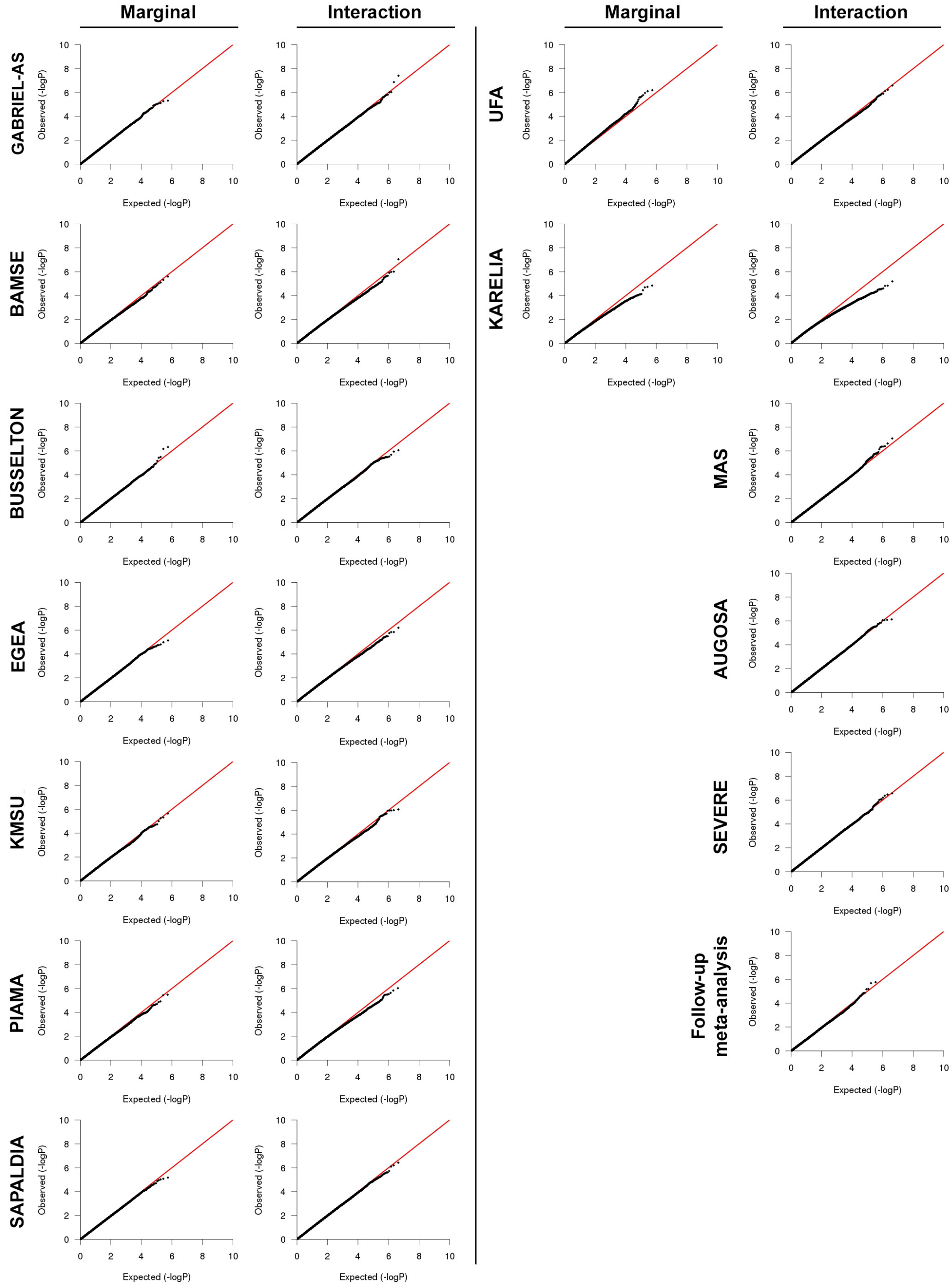


Marginal effects p-values were obtained via logistic regression in PLINK, for all SNPs that passed quality control (case-control studies only). To obtain interaction p-values in all studies except the follow-up meta-analysis, a random selection of 1% of all SNPs with MAF >= 0.20 was obtained, and all pairwise interactions among these SNPs were evaluated (for case-control studies, this included approximately $N = 4.5 \times 10^6$ interactions; for case-only studies, only inter-chromosomal interactions were included, resulting in approximately $N = 4 \times 10^6$ interactions). For case-control studies, the `--epistasis` function in PLINK was used to determine interaction p-values; for case-only studies, the `-afe-co-only` function in CASSI was used. The QQ plot for the follow-up meta-analysis included all interaction meta-analysis p-values (N = 345,034).

**Table S1. Subject quality control.**

| Sub-study | N, available | Excluded, subject call rate < 95% | Excluded, ambiguous SNP-sex | Excluded, relatedness; pi-hat >= 0.20 | Excluded, PCA outliers | N, final |
|---|---|---|---|---|---|---|
| AUGOSA | 688 | 34 | 0 | 9 | 20 | 625 |
| BAMSE | 505 | 0 | 0 | 4 | 40 | 461 |
| BUSSELTON | 1,468 | 6 | 4 | 170 | 83 | 1,205 |
| EGEA | 587 | 0 | 4 | 0* | 19 | 564 |
| GABRIEL-AS | 1,708 | 14 | 2 | 49 | 18 | 1,625 |
| KARELIA | 150 | 1 | 0 | 18 | 6 | 125 |
| KMSU | 585 | 2 | 1 | 20 | 16 | 546 |
| MAS | 183 | 1 | 0 | 1 | 8 | 173 |
| PIAMA | 404 | 1 | 0 | 0 | 42 | 361 |
| SAPALDIA | 1,612 | 6 | 2 | 55 | 88 | 1,461 |
| SEVERE | 348 | 4 | 3 | 6 | 45 | 290 |
| UFA | 707 | 15 | 1 | 6 | 19 | 666 |
| *Total* | *8,945* | *84* | *17* | *338* | *404* | *8,102* |

*: For the EGEA sub-study, non-founders were excluded prior to any of these quality control evaluations. The counts of subjects removed at each quality control step are mutually exclusive, since the steps were applied sequentially.

**Table S2. SNP quality control.**

| Sub-study | N, available | Excluded, could not map to hg19 | Excluded, SNP call rate < 98% | Excluded, HWE* | N, final |
|---|---|---|---|---|---|
| AUGOSA | 582,892 | 4,921 | 35,986 | 103 | 541,882 |
| BAMSE | 582,892 | 4,921 | 11,731 | 116 | 566,124 |
| BUSSELTON | 582,892 | 4,921 | 12,065 | 529 | 565,377 |
| EGEA | 582,892 | 4,921 | 6,938 | 286 | 570,747 |
| GABRIEL-AS | 582,892 | 4,921 | 16,723 | 532 | 560,716 |
| KARELIA | 582,892 | 4,921 | 25,221 | 28 | 552,722 |
| KMSU | 582,892 | 4,921 | 15,982 | 114 | 561,875 |
| MAS | 582,892 | 4,921 | 8,234 | 5 | 569,732 |
| PIAMA | 582,892 | 4,921 | 27,599 | 106 | 550,266 |
| SAPALDIA | 582,892 | 4,921 | 19,088 | 680 | 558,203 |
| SEVERE | 582,892 | 4,921 | 19,938 | 28 | 558,005 |
| UFA | 582,892 | 4,921 | 21,811 | 208 | 555,952 |
| *Median* | *582,892* | *4,921* | *17,906* | *115* | *559,460* |

*: Based on tests for deviation from Hardy-Weinberg equilibrium, using an exact test. For case-control studies, the threshold was HWE p-value < $10^{-4}$ in controls. For case-only studies, the threshold was HWE p-value < $10^{-7}$ in cases. The counts of SNPs removed at each quality control step are mutually exclusive, since the steps were applied sequentially. Note that "N, final" counts are *not* equal to the final numbers of SNPs that was actually analyzed; rather, these are the total number of SNPs *available* for possible analysis.

**Table S3. Final subject characteristics.**

| Sub-study | All subjects | Cases | | Controls | | Males | | Females | |
|---|---|---|---|---|---|---|---|---|---|
| | N | N | % | N | % | N | % | N | % |
| AUGOSA | 625 | 625 | 100 | 0 | 0 | 203 | 32.5 | 422 | 67.5 |
| BAMSE | 461 | 226 | 49.0 | 235 | 51.0 | 253 | 54.9 | 208 | 45.1 |
| BUSSELTON | 1,205 | 520 | 43.2 | 685 | 56.8 | 508 | 42.2 | 697 | 57.8 |
| EGEA | 564 | 120 | 21.3 | 444 | 78.7 | 287 | 50.9 | 277 | 49.1 |
| GABRIEL-AS | 1,625 | 802 | 49.4 | 823 | 50.6 | 932 | 57.4 | 693 | 42.6 |
| KARELIA | 125 | 57 | 45.6 | 68 | 54.4 | 41 | 32.8 | 84 | 67.2 |
| KMSU | 546 | 285 | 52.2 | 261 | 47.8 | 268 | 49.1 | 278 | 50.9 |
| MAS | 173 | 173 | 100 | 0 | 0 | 98 | 56.6 | 75 | 43.4 |
| PIAMA | 361 | 174 | 48.2 | 187 | 51.8 | 186 | 51.5 | 175 | 48.5 |
| SAPALDIA | 1,461 | 581 | 39.8 | 880 | 60.2 | 722 | 49.4 | 739 | 50.6 |
| SEVERE | 290 | 290 | 100 | 0 | 0 | 111 | 38.3 | 179 | 61.7 |
| UFA | 666 | 333 | 50.0 | 333 | 50.0 | 409 | 61.4 | 257 | 38.6 |
| *Total* | *8,102* | *4,186* | *51.7* | *3,916* | *48.3* | *4,018* | *49.6* | *4,084* | *50.4* |

**Table S4. Power analysis for the current study.**

| | Interaction Odds Ratio | α = $10^{-5}$ MAF (SNP 1 × SNP 2) 0.1 × 0.1 | 0.2 × 0.2 | 0.3 × 0.3 | 0.4 × 0.4 |
|---|---|---|---|---|---|
| **Screening analysis** Cases = 802 Controls = 823 | 0.33 | 0.09 | 0.87 | 0.99 | 0.99 |
| | 0.40 | 0.04 | 0.65 | 0.98 | 0.99 |
| | 0.50 | 0.01 | 0.28 | 0.76 | 0.94 |
| | 0.57 | 0.00 | 0.11 | 0.44 | 0.72 |
| | 0.67 | 0.00 | 0.02 | 0.10 | 0.23 |
| | 1.50 | 0.00 | 0.05 | 0.18 | 0.30 |
| | 1.75 | 0.01 | 0.27 | 0.65 | 0.79 |
| | 2.00 | 0.05 | 0.62 | 0.92 | 0.96 |
| | 2.50 | 0.24 | 0.96 | 0.99 | 0.99 |
| | 3.00 | 0.52 | 0.99 | 0.99 | 0.99 |

| | Interaction Odds Ratio | α = $1.45 \times 10^{-7}$ MAF (SNP 1 × SNP 2) 0.1 × 0.1 | 0.2 × 0.2 | 0.3 × 0.3 | 0.4 × 0.4 |
|---|---|---|---|---|---|
| **Follow-up Analysis *** Cases = 2,239 Controls = 3,025 | 0.33 | 0.99 | 0.99 | 0.99 | 0.99 |
| | 0.40 | 0.30 | 0.99 | 0.99 | 0.99 |
| | 0.50 | 0.07 | 0.93 | 0.99 | 0.99 |
| | 0.57 | 0.02 | 0.66 | 0.99 | 0.99 |
| | 0.67 | 0.00 | 0.14 | 0.63 | 0.89 |
| | 1.50 | 0.01 | 0.37 | 0.85 | 0.95 |
| | 1.75 | 0.10 | 0.95 | 0.99 | 0.99 |
| | 2.00 | 0.41 | 0.99 | 0.99 | 0.99 |
| | 2.50 | 0.93 | 0.99 | 0.99 | 0.99 |
| | 3.00 | 0.99 | 0.99 | 0.99 | 0.99 |

Power was estimated for pairwise interactions using Quanto version 1.2 (http://biostats.usc.edu/software) for a type I error (α) of either $10^{-5}$ (threshold for inclusion from the screening analysis) or $1.45 \times 10^{-7}$ (significance threshold for the follow-up analysis), as indicated. Shaded numbers indicate the power estimates; green shading indicates power estimates ≥ 0.80. Sample sizes for cases and controls are indicated in the leftmost column. Population risk: 0.075; main effect odds ratio (OR) for each gene: 1.00. Both SNPs are assumed to have the same minor allele frequency (MAF), as indicated. *: For the follow-up analysis, power is estimated assuming a single study equivalent in size to the sum of all cases and controls from the various follow-up sub-studies. As such, these estimates do not take into account between-study heterogeneity, which would reduce the actual power.

**Table S5. The top 10 most significant interactions in the follow-up analysis.**

| SNP 1 | | SNP 2 | | Screening | | Follow-up | | | | | | | | | | | | | | | | | | | |
|---|---|---|---|---|---|---|---|---|---|---|---|---|---|---|---|---|---|---|---|---|---|---|---|---|---|
| | | | | GABRIEL-AS | | BAMSE | | BUSSELTON | | EGEA | | KMSU | | PIAMA | | SAPALDIA | | UFA | | Meta-analysis | | | |
| | | | | OR | p | OR | p | OR | p | OR | p | OR | p | OR | p | OR | p | OR | p | OR | p | $I^2$ | Q |
| rs11684871 G/A | 2 | rs910652 T/C | 20 | 0.54 (0.42, 0.70) | 1.51 ×10-6 | 2.05 (1.26, 3.32) | 3.68 ×10-3 | 1.35 (1.03, 1.78) | 2.83 ×10-2 | 1.17 (0.68, 2.00) | 5.69 ×10-1 | 1.01 (0.66, 1.55) | 9.66 ×10-1 | 1.46 (0.90, 2.37) | 1.25 ×10-1 | 1.30 (1.01, 1.69) | 4.50 ×10-2 | 2.03 (1.35, 3.03) | 5.99 ×10-4 | 1.40 (1.22, 1.61) | 1.58 ×10-6 | 30.8 | 0.193 |
| rs11184943 C/T | 1 | rs1966938 A/C | 5 | 1.61 (1.31, 1.98) | 7.66 ×10-6 | 1.30 (0.86, 1.99) | 2.18 ×10-1 | 1.41 (1.09, 1.82) | 8.80 ×10-3 | 1.82 (1.14, 2.90) | 1.16 ×10-2 | 1.31 (0.87, 1.95) | 1.92 ×10-1 | 0.87 (0.53, 1.42) | 5.69 ×10-1 | 1.37 (1.09, 1.71) | 6.37 ×10-3 | 1.43 (0.97, 2.12) | 7.17 ×10-2 | 1.36 (1.20, 1.54) | 2.10 ×10-6 | 0.0 | 0.549 |
| rs3753394 C/T | 1 | rs16963466 C/T | 15 | 1.69 (1.35, 2.13) | 6.46 ×10-6 | 0.79 (0.47, 1.32) | 3.68 ×10-1 | 0.76 (0.59, 0.98) | 3.11 ×10-2 | 1.13 (0.72, 1.77) | 6.04 ×10-1 | 0.70 (0.48, 1.03) | 7.09 ×10-2 | 0.44 (0.25, 0.78) | 4.48 ×10-3 | 0.71 (0.56, 0.90) | 4.83 ×10-3 | 0.77 (0.55, 1.09) | 1.37 ×10-1 | 0.75 (0.66, 0.85) | 6.46 ×10-6 | 12.8 | 0.332 |
| rs3753394 C/T | 1 | rs7162621 A/G | 15 | 1.72 (1.37, 2.17) | 3.36 ×10-6 | 0.79 (0.47, 1.32) | 3.68 ×10-1 | 0.77 (0.60, 0.99) | 3.83 ×10-2 | 1.16 (0.74, 1.82) | 5.30 ×10-1 | 0.71 (0.49, 1.04) | 7.52 ×10-2 | 0.45 (0.25, 0.79) | 5.10 ×10-3 | 0.71 (0.56, 0.90) | 4.22 ×10-3 | 0.76 (0.54, 1.06) | 1.02 ×10-1 | 0.75 (0.66, 0.85) | 6.84 ×10-6 | 15.9 | 0.309 |
| rs7524009 G/A | 1 | rs4121621 A/G | 18 | 0.64 (0.52, 0.78) | 7.03 ×10-6 | 0.98 (0.67, 1.44) | 9.35 ×10-1 | 0.74 (0.58, 0.93) | 1.01 ×10-2 | 0.69 (0.44, 1.08) | 1.05 ×10-1 | 0.92 (0.65, 1.30) | 6.31 ×10-1 | N/A | N/A | 0.72 (0.58, 0.89) | 2.48 ×10-3 | 0.69 (0.48, 0.99) | 4.65 ×10-2 | 0.76 (0.68, 0.86) | 1.35 ×10-5 | 0.0 | 0.600 |
| rs3753394 C/T | 1 | rs1453950 G/A | 15 | 1.70 (1.35, 2.13) | 6.13 ×10-6 | 0.79 (0.47, 1.32) | 3.68 ×10-1 | 0.76 (0.59, 0.98) | 3.30 ×10-2 | 1.13 (0.72, 1.76) | 6.00 ×10-1 | 0.71 (0.49, 1.04) | 7.90 ×10-2 | 0.50 (0.29, 0.86) | 1.24 ×10-2 | 0.73 (0.57, 0.92) | 7.74 ×10-3 | 0.77 (0.55, 1.08) | 1.29 ×10-1 | 0.75 (0.66, 0.86) | 1.43 ×10-5 | 0.0 | 0.476 |
| rs7949405 A/C | 11 | rs2050095 T/C | 20 | 1.72 (1.36, 2.18) | 7.09 ×10-6 | 1.54 (1.04, 2.27) | 2.94 ×10-2 | 1.10 (0.86, 1.42) | 4.46 ×10-1 | 1.37 (0.85, 2.20) | 1.95 ×10-1 | 1.48 (1.00, 2.19) | 4.94 ×10-2 | 2.26 (1.36, 3.75) | 1.55 ×10-3 | 1.20 (0.94, 1.52) | 1.40 ×10-1 | 1.37 (0.98, 1.90) | 6.35 ×10-2 | 1.32 (1.16, 1.49) | 1.60 ×10-5 | 23.9 | 0.247 |
| rs12401360 A/G | 1 | rs1004704 C/T | 16 | 1.91 (1.44, 2.52) | 6.39 ×10-6 | 1.04 (0.65, 1.66) | 8.79 ×10-1 | 0.69 (0.52, 0.91) | 8.39 ×10-3 | 0.80 (0.44, 1.45) | 4.63 ×10-1 | 0.60 (0.38, 0.94) | 2.42 ×10-2 | 1.17 (0.63, 2.17) | 6.27 ×10-1 | 0.76 (0.58, 1.01) | 5.47 ×10-2 | 0.54 (0.37, 0.80) | 2.12 ×10-3 | 0.73 (0.63, 0.84) | 1.96 ×10-5 | 22.4 | 0.258 |
| rs4907013 A/G | 1 | rs1968586 C/T | 8 | 0.60 (0.48, 0.76) | 9.99 ×10-6 | 1.28 (0.87, 1.88) | 2.13 ×10-1 | 1.44 (1.12, 1.86) | 4.33 ×10-3 | 1.24 (0.78, 1.97) | 3.71 ×10-1 | 1.10 (0.77, 1.56) | 5.97 ×10-1 | 1.70 (1.04, 2.76) | 3.41 ×10-2 | 1.29 (1.01, 1.65) | 3.79 ×10-2 | 1.20 (0.86, 1.68) | 2.75 ×10-1 | 1.30 (1.15, 1.48) | 2.42 ×10-5 | 0.0 | 0.817 |
| rs2481672 G/A | 1 | rs6781598 C/T | 3 | 1.82 (1.40, 2.37) | 7.29 ×10-6 | 1.94 (1.16, 3.26) | 1.22 ×10-2 | 1.48 (1.08, 2.01) | 1.37 ×10-2 | 1.10 (0.64, 1.90) | 7.35 ×10-1 | 1.64 (1.07, 2.52) | 2.32 ×10-2 | N/A | N/A | 1.39 (1.04, 1.85) | 2.37 ×10-2 | 1.03 (0.67, 1.58) | 9.10 ×10-1 | 1.40 (1.20, 1.64) | 2.45 ×10-5 | 0.0 | 0.422 |

**SNP1** and **SNP2**: RSID, chromosome number, and alleles (major/minor) are listed for the SNPs involved in each respective interaction. **OR**: interaction odds ratio, with 95% confidence interval. **p**: p-value for a test of the null hypothesis that the interaction parameter is equal to zero. P-values highlighted in green are those with a value less than 0.05. **Meta-analysis**: meta-analysis of all follow-up studies (N=7 studies possible). $\mathbf{I^2}$: percentage of effect estimate variability due to heterogeneity between studies. **Q**: p-value from a Q test for heterogeneity. **N/A**: could not be estimated because one or both SNPs failed quality control in the indicated sub-study; in such cases, the sub-study was excluded from the meta-analysis.

**Table S6. Characteristics of rs910652 and rs11684871 (SNPs involved in the most significant interaction in the follow-up analysis).**

| | **rs910652**<br>Pos.: chr. 20: 3,727,970<br>Alleles (major/minor): T/C | | **rs11684871**<br>Pos.: chr. 2: 121,510,259<br>Alleles (major/minor): G/A | | Joint call count | **rs910652**<br>Marginal effect (C allele) | | **rs11684871**<br>Marginal effect (A allele) | |
|---|---|---|---|---|---|---|---|---|---|
| | MAF | HWE p-value | MAF | HWE p-value | | OR | p-value | OR | p-value |
| GABRIEL-AS | 0.323 | 0.750 | 0.255 | 0.315 | 1,620 / 1,625 | 1.05 (0.90, 1.21) | 0.545 | 0.93 (0.79, 1.09) | 0.361 |
| BAMSE | 0.300 | 0.641 | 0.294 | 0.635 | 458 / 461 | 0.90 (0.67, 1.20) | 0.472 | 0.83 (0.61, 1.11) | 0.211 |
| BUSSELTON | 0.345 | 0.933 | 0.271 | 1.000 | 1,204 / 1,205 | 0.96 (0.82, 1.14) | 0.659 | 0.96 (0.80, 1.15) | 0.671 |
| EGEA | 0.327 | 0.106 | 0.227 | 0.344 | 564 / 564 | 0.83 (0.61, 1.12) | 0.217 | 0.96 (0.67, 1.37) | 0.822 |
| KMSU | 0.276 | 0.163 | 0.297 | 0.882 | 544 / 546 | 1.03 (0.79, 1.35) | 0.827 | 0.96 (0.74, 1.25) | 0.769 |
| PIAMA | 0.309 | 0.492 | 0.267 | 0.711 | 360 / 361 | 1.09 (0.81, 1.48) | 0.558 | 1.09 (0.79, 1.50) | 0.611 |
| SAPALDIA | 0.316 | 1.000 | 0.236 | 0.349 | 1,452 / 1,461 | 1.03 (0.88, 1.21) | 0.709 | 0.97 (0.82, 1.15) | 0.735 |
| UFA | 0.291 | 0.894 | 0.267 | 0.486 | 666 / 666 | 0.96 (0.75, 1.22) | 0.714 | 0.92 (0.72, 1.18) | 0.523 |
| KARELIA | 0.257 | 0.117 | 0.231 | 0.314 | 124 / 125 | 0.87 (0.51, 1.50) | 0.618 | 0.85 (0.47, 1.54) | 0.584 |
| | | | | | Meta-analysis: | 0.99 (0.92, 1.07) | 0.860 | 0.95 (0.88, 1.02) | 0.164 |
| | | | | | Heterogeneity: | $I^2$ = 0%; Q = 0.900 | | $I^2$ = 0%; Q = 0.985 | |
| MAS | 0.327 * | 0.166 * | 0.263 * | 0.845 * | 173 / 173 | N/A | | | |
| AUGOSA | 0.336 * | 0.788 * | 0.250 * | 1.000 * | 625 / 625 | N/A | | | |
| SEVERE | 0.338 * | 0.602 * | 0.217 * | 0.864 * | 290 / 290 | N/A | | | |

**Pos.**: chromosome number and base pair position, in hg19 reference genome coordinates. **MAF**: minor allele frequency. **HWE p-value**: p-value for the Hardy-Weinberg equilibrium test. Both MAF and HWE were estimated among controls only, except for sub-studies denoted by an asterisk (*), for which the estimates were made within cases (since controls were not available for MAS, AUGOSA, and SEVERE). **Joint call count**: number of subjects for whom genotypes of both SNPs were available (numerator), over the total number of subjects (denominator). **Marginal effect, OR**: Marginal effect odds ratios (OR) for asthma, with 95% confidence interval, for each SNP considered individually in an additive model (via logistic regression). The minor alleles were the non-reference alleles. **Marginal effect, p-value**: p-value for the test of the null hypothesis that the marginal effect parameter is equal to zero. **Meta-analysis**: fixed effects meta-analysis results of marginal effects across all case-control studies (N=9 studies). **$I^2$**: percentage of effect estimate variability due to heterogeneity between studies. **Q**: p-value from a Q test for heterogeneity. **N/A**: could not estimate marginal effects, since controls were not available.

**Table S7. Characteristics of rs1018491 and rs67959717 (SNPs involved in the most significant interaction in the surrounding region of 20p13 and 2q14).**

| | **rs1018491** Pos.: chr. 20: 3,701,132 Alleles (major/minor): T/C | | **rs67959717** Pos.: chr. 2: 121,516,109 Alleles (major/minor): G/A | | | | Joint call count | **rs1018491** | | **rs67959717** | |
|---|---|---|---|---|---|---|---|---|---|---|---|
| | | | | | | | | Marginal effect (C allele) | | Marginal effect (A allele) | |
| | MAF | HWE p-value | MAF | Conc. | Info | Cert. | | OR | p-value | OR | p-value |
| GABRIEL-AS | 0.378 | 0.411 | 0.255 | 95.4 | 0.978 | 0.991 | 1,571 / 1,625 | 1.06 (0.92, 1.23) | 0.417 | 0.92 (0.79, 1.09) | 0.334 |
| BAMSE | 0.372 | 0.889 | 0.296 | 96.0 | 0.983 | 0.991 | 450 / 461 | 0.96 (0.73, 1.25) | 0.750 | 0.84 (0.63, 1.13) | 0.251 |
| BUSSELTON | 0.381 | 0.808 | 0.273 | 95.8 | 0.979 | 0.991 | 1,181 / 1,205 | 0.93 (0.78, 1.09) | 0.362 | 0.95 (0.79, 1.14) | 0.600 |
| EGEA | 0.412 | 0.282 | 0.224 | 95.0 | 0.976 | 0.990 | 550 / 564 | 0.92 (0.70, 1.23) | 0.578 | 0.99 (0.69, 1.41) | 0.952 |
| KMSU | 0.326 | 0.675 | 0.301 | 95.7 | 0.976 | 0.987 | 526 / 546 | 1.08 (0.84, 1.39) | 0.544 | 0.96 (0.74, 1.25) | 0.755 |
| PIAMA | 0.395 | 0.356 | 0.269 | 94.8 | 0.982 | 0.991 | 351 / 361 | 0.92 (0.68, 1.25) | 0.604 | 1.08 (0.78, 1.49) | 0.648 |
| SAPALDIA | 0.381 | 0.351 | 0.236 | 95.2 | 0.974 | 0.989 | 1,405 / 1,461 | 1.12 (0.96, 1.31) | 0.137 | 0.98 (0.82, 1.17) | 0.832 |
| UFA | 0.348 | 0.115 | 0.267 | 95.9 | 0.986 | 0.994 | 654 / 666 | 1.02 (0.82, 1.27) | 0.868 | 0.92 (0.71, 1.18) | 0.516 |
| KARELIA | 0.287 | 1.000 | 0.272 | 97.0 | 0.984 | 0.993 | 122 / 125 | 1.40 (0.82, 2.39) | 0.222 | 0.65 (0.35, 1.19) | 0.161 |
| | | | | | | | Meta-analysis: | 1.03 (0.96, 1.10) | 0.464 | 0.94 (0.87, 1.02) | 0.141 |
| | | | | | | | Heterogeneity: | $I^2$ = 0%; Q = 0.678 | | $I^2$ = 0%; Q = 0.928 | |
| MAS | 0.364 * | 0.623 * | 0.263 * | 95.8 | 0.988 | 0.995 | 171 / 173 | N/A | | | |
| AUGOSA | 0.374 * | 0.864 * | 0.256 * | 95.4 | 0.981 | 0.992 | 608 / 625 | N/A | | | |
| SEVERE | 0.381 * | 0.458 * | 0.218 * | 95.3 | 0.984 | 0.993 | 285 / 290 | N/A | | | |

Rs1018491 was directly genotyped, while rs67959717 was imputed, for all sub-studies. **Pos.**: chromosome number and base pair position, in hg19 reference genome coordinates. **MAF**: minor allele frequency. **Conc.**: concordance, a quality metric for the imputation interval; the percentage of directly genotyped SNPs within the imputation interval whose imputed genotypes matched their true genotypes. **Info**: an imputation quality metric for the SNP; see (E16). **Certainty**: an imputation quality metric for the SNP; the average probability of the most probable genotypes. **HWE p-value**: p-value for the Hardy-Weinberg equilibrium test. Both MAF and HWE were estimated among controls only, except for sub-studies denoted by an asterisk (*****), for which the estimates were made within cases (since controls were not available for MAS, AUGOSA, and SEVERE). **Joint call count**: number of subjects for whom genotypes of both SNPs were available (numerator), over the total number of subjects (denominator). For rs67959717, the SNP was considered called if its highest probability genotype had a probability ≥ 0.90. **Marginal effect, OR**: Marginal effect odds ratios (OR) for asthma, with 95% confidence interval, for each SNP considered individually in an additive model (via logistic regression). The minor alleles were the non-reference alleles. **Marginal effect, p-value**: p-value for the test of the null hypothesis that the marginal effect parameter is equal to zero. **Meta-analysis**: fixed effects meta-analysis results of marginal effects across all case-control studies (N=9 studies). **$I^2$**: percentage of effect estimate variability due to heterogeneity between studies. **Q**: p-value from a Q test for heterogeneity. **N/A**: could not estimate marginal effects, since controls were not available.
